# Bovine Babesiosis in Turkey: Impact, Current Gaps, and Opportunities for Intervention

**DOI:** 10.3390/pathogens9121041

**Published:** 2020-12-11

**Authors:** Sezayi Ozubek, Reginaldo G. Bastos, Heba F. Alzan, Abdullah Inci, Munir Aktas, Carlos E. Suarez

**Affiliations:** 1 Department of Veterinary Microbiology and Pathology, Washington State University, Pullman, WA 99164-7040, USA; sezayi.ozubek@wsu.edu (S.O.); reginaldo_bastos@wsu.edu (R.G.B.); heba.alzan@wsu.edu (H.F.A.); 2 Department of Parasitology, Faculty of Veterinary Medicine, University of Firat, 23119 Elazig, Turkey; maktas@firat.edu.tr; 3 Parasitology and Animal Diseases Department, National Research Center, Dokki, Giza 12622, Egypt; 4 Department of Parasitology, Faculty of Veterinary Medicine, Erciyes University, 38039 Kayseri, Turkey; ainci@erciyes.edu.tr; 5 Vectors and Vector-Borne Diseases Implementation and Research Center, Erciyes University, 38039 Kayseri, Turkey; 6 Animal Disease Research Unit, USDA Agricultural Research Service, Pullman, WA 99164-6630, USA

**Keywords:** bovine babesiosis, *Babesia*, bovine, cattle, cattle industry in Turkey

## Abstract

Bovine babesiosis is a global tick-borne disease that causes important cattle losses and has potential zoonotic implications. The impact of bovine babesiosis in Turkey remains poorly characterized, but several *Babesia* spp., including *B. bovis*, *B. bigemina*, and *B. divergens*, among others and competent tick vectors, except *Rhipicephalus microplus*, have been recently identified in the country. Bovine babesiosis has been reported in all provinces but is more prevalent in central and highly humid areas in low and medium altitude regions of the country housing approximately 70% of the cattle population. Current control measures include acaricides and babesicidal drugs, but not live vaccines. Despite the perceived relevant impact of bovine babesiosis in Turkey, basic research programs focused on developing in vitro cultures of parasites, point-of-care diagnostic methods, vaccine development, “omics” analysis, and gene manipulation techniques of local *Babesia* strains are scarce. Additionally, no effective and coordinated control efforts managed by a central animal health authority have been established to date. Development of state-of-the-art research programs in bovine babesiosis to address current gaps in knowledge and implementation of long-term plans to control the disease will surely result in important economic, nutritional, and public health benefits for the country and the region.

## 1. Introduction

An important chapter on animal infectious diseases began in 1888 with the sudden deaths of thousands of cows in Romania [1]. At the time, Victor Babes associated the animal deaths with an intraerythrocytic organism, which he named as “haematococcus.” These organisms were later identified as protozoan parasites, and renamed *Babesia* to honor its discoverer [2]. A few years later, two researchers in the US, Smith and Kilbourne, found out that *Babesia* parasites were transmitted by Ixodid ticks, demonstrating for the first time transmission of a parasite by an arthropod vector [3]. Follow up studies have characterized several species of *Babesia* as tick-transmitted apicomplexan protozoan hemoparasites with veterinary and human importance, and great economic impact worldwide [4]. Babesiosis is a disease that affects many vertebrate hosts, from humans to bats, as well as farm animals, such as cattle, horses, and small ruminants, and companion animals [5]. There are more than 100 *Babesia* species reported so far, with different host specificities [6]. Here, we focus on bovine babesiosis, a disease with a particular large impact on cattle worldwide. In addition to major economic losses derived from death of animals and decreased production of meat and dairy products as a result of bovine babesiosis, there are other important costs associated with tick control, diagnosis, and treatments required to prevent the disease. Despite the importance, there is no reliable specific quantification of the impact of bovine babesiosis at the global scale, but only independent regional assessments performed in individual countries, such as Brazil, Argentina, Australia, among others, and the estimated losses are in the order of hundreds of millions of US dollars per year.

*Babesia* parasites have a complex lifecycle that includes the development of asexual stages in mammalian hosts and sexual stages inside their definitive tick vectors. Two characteristics that define *sensu stricto Babesia* parasites are their ability to be transmitted transovarially by tick vectors and exclusively infect red blood cells (RBC) in their vertebrate host. These aspects are particularly important for *B. bovis*, *B. bigemina*, and *B. divergens*, the major causative agents of bovine babesiosis [6,7].

Growth of asexual stages of *Babesia* parasites inside the vertebrate host RBC causes severe intravascular hemolytic anemia, which is a pathognomonic sign of the acute disease and highly debilitating for the host. Additionally, fever, prostration, abortion, and temporary infertility are also common clinical findings during acute infection. Hemoglobinuria is also usually present at the peak of the hemolytic crisis in *B. bigemina* or *B. divergens* infection and in late stages of the disease caused by *B. bovis*. In addition, residues and toxic metabolites released as a result of the infection and RBC destruction can negatively affect host organ systems [6,7,8]. Moreover, *B. bovis* has the unique ability to evade the cattle immune system by expressing proteins that facilitate cytoadhesion of infected RBC to capillaries, such as in the brain, causing neurological symptoms and generalized organ failure, a feature that results in increased virulence. Altogether, these pathological mechanisms frequently lead to rapid death of cattle during the acute stage of the disease, especially when affecting adult naïve animals.

Upon infection, the immune system of the host responds differentially, depending on the age of the animals. While young animals, less than seven months old, are frequently able to control severe acute babesiosis and can survive re-exposures to the parasites, older than one-year-old animals often succumb rapidly to infection. Features associated with resistance in young animals include early and strong activation of the innate and adaptive immune effectors. Briefly, the parasite expresses molecules able to bind pathogen associated molecular patterns (PAMPs) receptors expressed on the surface of dendritic cells (DC), macrophages, neutrophils, and monocytes, especially TLR9 [9,10], and an immune response is initiated. Cytokines, such as IL-1β, TNF-α, and IL-12, and nitric oxide (NO) are released from monocytes and neutrophils, and chemokines attract immature DC to the site of infection, especially at the spleen. These stimulate natural killer cells (NKs) that release early IFNγ. The mature DC migrating to the spleen presents *Babesia* antigens to naive T cells. Spleen macrophages are activated by IFNγ, phagocytize infected RBC, and kill the parasites by releasing reactive nitrogen and oxygen intermediates. In turn, cytokines, such as 1L-1β, IL-12, and TNF-α, released from activated macrophages inhibit the growth of *B. bovis*. Activated CD4^+^ T cells and specific B-cell producing antibodies are also important in maintaining immunity and overcoming the infection [11,12,13,14]. Despite mounting protective adaptive immune responses, animals that survive acute babesiosis develop persistent infection, which allows transmission and perpetuation of *Babesia* parasites in endemic areas. These areas usually have elevated prevalence of bovine babesiosis but low numbers of clinical cases due to the establishment of endemic stability, a condition of herd immunity that develops when more than 75% of the animals have acquired protective immunity by exposure to the parasite before one year of age, when animals are less susceptible to the parasites. A highly unstable state may occur, in contrast, when less than 30% of the herd is naïve for the disease [15,16,17].

*B. bovis* and *B. bigemina*, which are transmitted by *Rhipicephalus* ticks, are the most important causative agents of bovine babesiosis in tropical and subtropical regions worldwide. In addition, *B. divergens* is another important *Babesia* species that is transmitted by *Ixodes* ticks and affects cattle in Europe and North Africa. Apart from its impact on bovines, *B. divergens* is especially important as a zoonotic pathogen implicated in human babesiosis in Europe [6,7,8,18]. Acute bovine babesiosis caused by *B. bovis* is often severe due to cytoadhesion of infected RBC in the lung, kidney, and brain capillaries, which leads to hypotension, respiratory stress syndrome, neurological symptoms, and death [8,19]. In contrast, *B. bigemina* induces massive hemolytic anemia without causing the symptoms associated with cytoadhesion [4,6,18]. Also of importance as causative agents of bovine babesiosis are *B. ovata*, *B. major*, *B. occultans*, and additional unclassified *Babesia* that are characterized by their low pathogenicity in cattle compared to *B. bovis*, *B. bigemina*, and *B. divergens* [8,20,21,22,23,24].

Numerous cases of bovine babesiosis involving most of these mentioned *Babesia* species have been reported throughout the years (from 1955 to 2020) in Turkey. Despite the overall economic importance of bovine babesiosis worldwide, only relatively few studies have addressed the impact of the disease on the cattle industry in this country, and updates on this important subject have also been overlooked in recent years. Therefore, the focus of this study is on compiling relevant studies to improve our understanding of the effect of bovine babesiosis on the Turkish cattle industry, with the aim of assessing the current status of the disease and identifying research and intervention gaps that can contribute to improved control of this disease. Here we cover not only the major causative agents *B. bovis*, *B. bigemina*, and *B. divergens*, but also less virulent species recently identified in Turkey, such as *B. major* and *B. occultans*, among others. In addition, we seek to stimulate basic and applied research on this field that is currently neglected with the expectation that this will result in substantial benefits on food production, animal health, economic growth, and human well-being in Turkey and surrounding countries. 

## 2. An Overview of the Cattle Industry in Turkey

Turkey is a transcontinental country located in the Northern hemisphere with a territory spanning the Anatolian peninsula in Western Asia and a small portion on the Balkan Peninsula in Southeastern Europe. Geographic coordinates of the country lie at latitude 39 and longitude 35. It is a peninsula with a strategic position as a land connection between Europe and Asia. Considering its location, the country has been also regarded as a natural bridge for transcontinental transmission of tick species and tick-borne diseases (TBD) [25]. Turkey covers an area of 783,582 km^2^ with a population of 82 million people. The country’s economy is based on modern industry, tourism, and trade, and is also heavily supported by the agricultural sector. Therefore, the presence of emerging tick populations and TBD can pose a serious risk to the cattle industry that may impact the overall economy of Turkey and neighboring countries [25,26]. 

Although the importance of cattle has been different in every society throughout the history of humanity, these animals have always had significant importance in several cultures and religions, and still remain an important economic asset in Turkey and worldwide. There are approximately 66 million farm animals in Turkey, and 27% of which (18 million) are cattle. In Turkey, milk production consisted of 90.5% cattle, 6.6% sheep, and 2.5% goat. As for meat production, 89.5% of the total meat produced in Turkey comes from cattle. According to a report from 2018, approximately 1.5 million live animals are imported to Turkey [27]. Some of these animals come from countries that are endemic for cattle ticks, bovine babesiosis, and other cattle TBD, such as Brazil, from where Turkey obtains 42% of its total imported animals [27,28]. Considering that cattle are a very important source of protein, especially meat and milk production, the cattle industry is a significant sector to secure food supply and sustain the economy in Turkey [27]. Assuring constant supply of cattle milk and meat requires keeping high animal sanitary standards and rational strategies for industry development. In addition, particular attention should be placed in controlling diseases that limit cattle production and may compromise public health. This should be extended to the potential introduction of additional animal health risk factors, such as foreign pathogenic organisms. Taking into consideration the social and economic importance of cattle in Turkey, we argue that the development of a national intensive research program on TBD, specifically in bovine babesiosis, and the implementation of informed animal health policies of disease control based on the state-of-the-art knowledge should be considered issues of crucial importance for the country.

## 3. Economic Impact of Bovine Babesiosis on the Cattle Industry in Turkey

It is estimated that more than 500 million cattle are at risk of babesiosis worldwide; therefore, this disease poses a major threat to animal health and human livelihood in areas where *Babesia* parasites and competent tick vectors are present [4]. As an attempt to contain such threat, a radical tick control campaign was launched in the US at the beginning of the 20th century, lasting 40 years and demanding the use of millions of taxpayer’s dollars. With this effective, but costly campaign, bovine babesiosis was eradicated in the US, and consequently approximately $3 billion US dollars annually were saved for the livestock industry [6,29,30]. Unfortunately, the success of this approach was not reproduced in other countries that also attempted similar tick eradication [31], and given a current scenario of increased acaricide resistance in ticks and climate change, among other factors, it is unlikely that this achievement can be duplicated elsewhere [30,31,32].

Annual economic losses due to bovine babesiosis and anaplasmosis in the world range from $16.9 million US dollars in Australia, $21.6 million US dollars in South Africa, and $57.2 million US dollars in China [8]. These losses are not only due to animal mortality, but also abortion, decrease in meat and milk production, and disease control costs (e.g., spraying, vaccination, disease treatments, professional veterinary support, and others). In addition, disease-related deaths are frequently observed in naïve cattle imported to regions with enzootic stability for bovine babesiosis [6,8,33], a factor that causes additional economic losses and complicates the attempts to carry out genetic improvement of herds. In this way, preventing clinical cases of bovine babesiosis by strategies based on maintaining enzootic stability may also interfere with the efforts to improve productivity, such as increased weight and milk production, heavily affecting the meat and dairy industries, respectively, which increases production costs [6,8,17]. 

Turkey’s geographic location and climatic conditions, in addition to the country’s animal management systems, encourage the occurrence of ticks and TBD [25,26]. The emergence of tick populations and TBD have increased around the globe in the recent years, including in Turkey [25]. Estimation of the amount of TBD drugs sale per year during the disease seasons indirectly shows the importance of these diseases on animal health and in the economy of Turkey [26]. The economic impact of topical theileriosis caused by *Theileria annulata*, a tick-borne parasite similar to *Babesia* sp., was estimated at a total annual loss of approximately 600,000 US dollars in Turkey [34]. However, despite being considered as a costly burden, the actual economic impact of bovine babesiosis on the cattle industry in Turkey remains largely unknown. Therefore, a well-designed national surveillance study to evaluate the real impact of the disease on the cattle industry in the country is urgently needed. 

## 4. Competent Tick Vectors for *Babesia* Parasites Identified in Turkey

Tick vectors are essential components for the completion of the lifecycle of *Babesia* parasites. Thus, competent ticks must provide the environment required for sexual reproduction, which occurs in their midgut, and for invasion of tick eggs by the kinete stage of parasites that circulates in the tick hemolymph, an event that ultimately guarantees transovarial transmission of *Babesia*. A large number of Ixodid tick species are listed as competent *Babesia* vectors in the literature [5]. Of these, 22 were confirmed vectors for 18 different *Babesia* species that infect livestock, companion animals, and humans [5]. Identification of pathogen DNA in adult ticks cannot be accepted alone as evidence of vector competence, and more detailed studies on tick-*Babesia* interactions are needed to establish the tick competence. Additionally, the presence of *Babesia* DNA in the salivary glands, eggs, and unfed larvae, though more convincing, also requires confirmation as a measure of tick competence [5]. *B. bovis* and *B. bigemina* are transmitted by *R. annulatus*, *R. microplus*, and *R. geigyi* ticks found in tropical and temperate regions of the world. *B. bigemina* can also be transmitted by *R. decoloratus* and *R. evertsi*, making it the most common *Babesia* species infecting cattle in Africa [8,35]. *B. divergens* are transmitted mainly by *I. ricinus*, which develops only in moisture-saturated microhabitats [19]. *B. occultans*, *B. major*, *B. orientalis*, and *B. ovata* are transmitted by *Hyalomma rufipes*, *Haemaphysalis punctata*, *R. haemaphysaloides*, and *Hae. longicornis*, respectively [5]. A summary of known competent tick vectors implicated in bovine babesiosis is shown in Table 1, where we highlight the species present in Turkey.

To date, *R. annulatus*, *R. bursa*, *R. turanicus*, *R. sanguineus*, *Hy. anatolicum*, *Hy. dromedari*, *Hy. detritum*, *Hy. excavatum*, *Hy. marginatum*, *Hy. rufipes*, *Hy. aegyptium*, *Dermacentor marginatus*, *D. niveus*, *I. ricinus*, and *Hae. parva* ticks have been reported infesting cattle in Turkey [25,46], and some of them were associated with transmission of cattle *Babesia* parasites (Table 1). In a study using ticks collected from cattle in the Black Sea region, *Babesia* parasites were reported in *Hy. marginatum*, *Hy. Excavatum*, and *R. turanicus* at the rates of 3.5%, 2.3%, and 6.6%, respectively [47]. *Babesia* sp. Kayseri 1, a novel parasite isolate, was identified in *Hy. marginatum* feeding on cattle in the Kayseri province located in Central Anatolia [48]. *B. bigemina* was also reported in unfed larvae from *R. annulatus* in this same province [48]. In another study in the same region, *B. bigemina* was found in tick populations of *R. annulatus*, *R. turanicus*, *Hy. marginatum*, and *Hy. Anatolicum*, whereas *B. bovis* positive samples were detected in *Hy. marginatum* ticks [49]. *B. occultans* was reported in *Hy. marginatum* and *R. turanicus*, as well as in their eggs, and thus, these findings suggest that this later tick can also be a competent vector for *B. occultans* [50]. In another study, *B. occultans* was identified in questing Hy. marginatum [51]; however, despite the findings, effective transmission of *Babesia* by these ticks remains to be demonstrated in Turkey. 

Collectively, currently available data indicate the presence and expansion of tick populations in Turkey. In addition, most of these tick species have been shown to be competent in transmitting *Babesia* parasites implicated in bovine babesiosis. Considering the current environmental changes and the importance of the cattle industry in Turkey, epidemiological and entomological studies focused on ticks associated with *Babesia* transmission are urgently needed in the country. Given the absolute dependence of ticks for parasite survival, identifying all competent vectors for *Babesia* species circulating in the country and a more complete understanding of the dynamics of the *Babesia*-tick interactions will be essential to achieve improved control of bovine babesiosis in Turkey.

## 5. Current Control Methods

Live, attenuated, blood based *Babesia* vaccines, combined with babesicidal drugs and strategies to control tick vectors, are commonly used to prevent and treat bovine babesiosis worldwide, especially in endemic areas. Here we present a brief description of these methods and their applications and importance for the control of bovine babesiosis in Turkey. 

### 5.1. Anti-Babesia Vaccines

Live vaccines based on attenuated parasites are widely used to control bovine babesiosis in many countries, such as Australia, Israel, and Argentina [8,10,17]. *B. bovis* attenuated vaccine strains are produced by quick serial passages of infected blood in splenectomized calves (ranging from 23–30 animals) [52]. In contrast, *B. bigemina* vaccine strains are produced by slow serial passages (3 to 16-week intervals) in intact spleen calves [53]. Attempts were also made to produce a live vaccine for *B. divergens* in splenectomized calves or intact gerbils. However, treatment with babesicidal drugs was needed to prevent clinical manifestations after vaccination [18], and due to its poor efficacy, this anti-*B. divergens* live vaccine is no longer in use [18]. The mechanisms involved in the process of attenuation of *Babesia* parasites during passages in calves still remain unknown, but recent genomic and transcriptomic comparative analyses among parental virulent and their derived attenuated vaccine strains have shed some light on the presence of differences in the patterns of expression of a limited number of genes [54]. For instance, expression of the spherical body protein 2t11 (SBP211t) was found upregulated in attenuated vaccine strains of *B. bovis* [55], suggesting that comparing the levels of expression of this gene among strains could be correlated with virulence. Hence, it would be of relevance to compare the pattern of expression of SBP2t11 among local parasite strains isolated in Turkey in order to select parasites that could be candidates for the development of live vaccines. 

Despite their relative efficacy, current live vaccines have several limitations, and their use for the control of bovine babesiosis is somehow considered a double-edged sword. On one side, live vaccines remain the most effective way of controlling acute bovine babesiosis, especially considering that there are no safer alternative subunit vaccines available against the disease. Additionally, a study performed in Argentina suggested that the systematic application of live *Babesia* vaccines in calves might render a benefit-cost ratio between 4.6 and 9.0 [56]. Ojeda et al. 2010 [57] also reported high levels of protection conferred by the vaccine (93%) and demonstrated a ratio of ill/healthy (vaccinated:non-vaccinated) animals of 1:6.5. On the other side of the sword, production of live vaccines is expensive and laborious, cold chain is needed, and contamination with infectious agents and reversion to virulence of vaccine strains remain a possibility. In addition, live vaccines may also lead to transmission of the vaccine strains by ticks, which can contribute to the generation of antigenic diversity among field strains and affect endemic stability. To prevent these problems, live vaccines lacking genes required for sexual reproduction of the parasite using transfection or gene editing technologies can be alternatively developed [58]. Other options available to overcome the various disadvantages of live vaccines are to develop formulations containing killed parasites, soluble parasite antigens (SPAs), and recombinant protective parasite antigens [59]. SPAs are released into peripheral blood during *Babesia* infection, and promising results in *B. canis* and *B. rodhaini* studies suggest that these antigens are potential candidates for developing similar vaccines against other *Babesia* species [60]. Thus, SPAs prepared from supernatants of in vitro cultures have shown promising results against *B. bigemina* [61], *B. canis* [62], *B. divergens* [63], and *B. orientalis* [64]. However, on the downside, it was found that vaccines based on *B. bovis* SPAs were more effective in protecting against challenge with homologous than heterologous strains, raising concerns about their use as universal vaccines. 

Although many studies have been conducted to develop subunit vaccines against babesiosis based on recombinant proteins, a commercial-grade product has still not been obtained so far. The use of eukaryotic instead of prokaryotic expression systems mixed with new-generation adjuvants has been recommended in order to increase vaccine efficacy [59]. Recombinant proteins can also be used in the development of transmission-blocking vaccines (TBV), based on parasite sexual stage proteins that are usually exclusively expressed in ticks [58,65,66]. Testing of candidate antigens for TBV against *Plasmodium* [67] have suggested the notion that such vaccines may also be efficient against *Babesia* [59]. Several previously characterized *B. bovis* sexual and tick-stage antigens, such as HAP2 [66], CCp1-3 [65], and 6 Cys [58,68], are also being currently considered as candidates for TBV. In addition, recently characterized *B. bigemina* sexual-stage antigens are also considered as possible candidates for the development of TBV [69,70]. A possible future effective vaccination strategy would be a combination of a TBV, which can block the development of sexual stage parasites in ticks to prevent parasite transmission in endemic regions, with a blood-stage live subunit vaccine to prevent acute babesiosis. Hence, this combination of blood and tick-stage antigens can provide effective protection while, at the same time, diminishing the load of *Babesia* circulating in tick vectors in endemic areas. Yet, these vaccines will still require the pending definition of protective and well-conserved blood and tick stage antigens. An important body of work has been also developed in the search of blood stage vaccine candidate antigens, using several approaches. Thus, the *B. bovis* RAP-1 [71], RRA [4], MSA-2 [72], TRAPs [73], and AMA-1 [74,75] proteins, among others, are currently being investigated as a component of subunit vaccines. It would be important to determine the degree of conservation of such candidate antigens in *B. bovis* strains isolated in Turkey as well as the presence of equivalent homologous proteins expressed by other cattle *Babesia* parasites common in this country. 

Live vaccines are not currently under production, and therefore, not being utilized in Turkey to prevent bovine babesiosis, and acaricides aimed at controlling ticks is the only method for the disease prevention in use in the country, as described in detail below. However, it needs to be considered that a *Babesia*-control strategy based solely on targeting vector control appears unrealistic due to multiple factors that include the constant emergence of acaricide resistant tick strains, the multiplicity of competent vectors for these parasites, global climate change that favors the geographic expansion of ticks, and the negative impact that acaricides may have on the environment, among others.

### 5.2. Anti-Tick Control Strategies

Acaricides have been applied for centuries to reduce the harmful effects of ticks and TBD on their vertebrate hosts. Although they have shown efficacy in controlling ticks, acaricides have numerous disadvantages, such as contamination of animal products and the environment. As a control strategy against babesiosis, acaricides are used to decrease or prevent tick infestation of susceptible hosts. However, with the gradual global emergence of acaricide-resistant ticks, the need for the development of new effective drugs and anti-tick vaccines are needed worldwide [17,76]. Yet, despite the implications derived from the emergence of acaricide resistant tick populations, such reports remain unavailable in Turkey. Currently, formamidines, synthetic pyrethroids, phenyl pirazoles, and macrocyclic lactones are used for tick control in the country [25]. The target site for organophosphates and carbamates are acetylcholinesterases that break down the neurotransmitter acetylcholine, while the targets for pyrethroids are voltage-gated Na^+^ channel regulatory proteins of the nerve membrane [77]. Alternatively, the use of anti-tick vaccines surge as an economical and environmentally sustainable approach. Two commercial anti-tick vaccines, TickGARD and Gavac, also known as Bovimune Ixovac in Mexico (http://www.lapisa.com), against *R. microplus* have been used around the world [78]. While apparently Gavac continues to be commercialized in Cuba (Heber Biotec S.A., Havana, Cuba), and more recently Bovimune Ixovac in Mexico (Lapisa, S.A. de C.V., La Piedad, Mexico), TickGARD is no longer commercially available [79]. These subunit anti-tick vaccines are based on the concealed tick midgut glycoprotein antigen Bm86, which has shown efficacy in reducing the number of engorged female ticks, their weight and fertility capacity, and also in decreasing larval infestation in subsequent tick generations. Cost-effectiveness analysis showed a 60% reduction in the number of acaricide treatments, together with the control of tick infestations and transmission of babesiosis, which resulted in savings of US $23.4 animal/year [80]. A comprehensive vaccination program in Venezuela reported that the Gavac™ vaccine reduced the use of chemical acaricides by 83.7%. The vaccine program was also reported to reduce 81.5% of the estimated cost of conventional chemical tick control procedures [81]. Despite their initially demonstrated efficacy, Bm86 vaccines did not show consistent protection when applied in diverse geographic locations around the world [82]. This lack of effectiveness may be in part due to antigenic polymorphisms, patterns of expression in the ticks, or intrinsic characteristics of the Bm86 antigen, such as conformation and post-translational modifications. It is important to highlight that none of these Bm86-based anti-tick vaccines are used or commercialized in Turkey. Additional tick antigens, such as Bm91 and Bm95 (alternative polymorphic versions of Bm86 isolated from an Argentinian *R. microplus* strain resistant to vaccination with Bm86), among others, have been characterized and also considered for vaccines [76,83]. Whether Bm86 vaccines or other vaccine based on Bm86 alternative antigens may contribute to tick control in Turkey remains unknown since none of them has been tested in a field trial and are unavailable for use in the country. Tick genome analysis, together with the integration of transcriptomics, proteomics, and metabolomics datasets will surely facilitate the identification of effective protective antigens. Recently, “omics” technologies have shown to be effective approaches for the characterization of tick-host-pathogen molecular interactions, and the potential for vaccine development has emerged. These vaccines should be designed considering tick vector species and susceptible hosts [84]. Studies have shown that the “vaccinomics” approach to select protective antigens (Silk and Subolesin) is valid, and it has been shown that vaccines based on tick proteins involved in vector-pathogen interactions can be used for controlling tick infestation and pathogen infection [85,86].

### 5.3. Babesia Drug-Control Strategies

Imidocarb dipropionate and diminazen acetate are the most common drugs used in the treatment of babesiosis in cattle and other animals. Despite their efficacy in controlling acute disease in some cases, these drugs are expensive and leave residual metabolites in milk and meat. Continuous or inappropriate use of these drugs can also lead to parasite resistance [87]. As a result of all these adversities, research efforts have been focused on developing new alternative, effective, and affordable drugs with low toxicity for the control of bovine babesiosis. It has been reported that strategies based on the combination of chemotherapeutics are significantly more effective at eliminating parasites compared to single-drug treatment, which also has the potential to induce greater parasite drug resistance [88]. In addition, combined chemotherapy decreases toxic side effects by reducing the dosages of individual medications (not reported for imidocarb and diminazen) [89]. In recent years, many drugs, such as nimbolide, gedunin, enoxacin, luteolin, pyronaridine tetraphosphate, nitidine chloride, camptothecin, tulathromycin, trifluralin, 17-DMAG, thymoquinone, clofazimine, carfilzomib, and doxorubicin hydrochloride, have been investigated in vitro against *Babesia* parasites [90,91,92,93,94,95,96,97,98,99]. Despite promising results, none of these drugs are used in the field as commercially available treatments for bovine babesiosis. 

Another important consideration that limits the use of chemotherapeutics, besides their elevated cost and the possible development of drug resistance, is their potential to interfere with the development of herd anti-*Babesia* immunity in endemic areas. Thus, even when this approach may be effective in the treatment of acute bovine babesiosis, the use of chemotherapeutics is not considered an ideal control method. Noteworthy, the only chemotherapeutic drug licensed for use in Turkey to treat bovine babesiosis is imidocarb dipropionate [100] complemented at times with supportive treatments, such as iron preparations, dextrose, B vitamins, and fluid replacement, which are especially important in acute severe cases.

Considering the current progresses and drawbacks associated with the available anti-*Babesia* vaccines, babesicidal drugs, and tick control measures, we suggest the design and implementation of pro-active national and international collaborative programs to assess the current situation, identify current gaps, and design improved control strategies. As an example, identification, characterization and integration of local strain of *Babesia* parasites that cause bovine babesiosis in Turkey in the development of novel control measures for the disease can greatly increment the preparedness of the country to adequately manage this important animal health concern.

## 6. Diagnosis of Bovine Babesiosis

Although clinical symptoms, season, and tick infestation may be suggestive of the occurrence of bovine babesiosis, confirmatory diagnosis using different techniques needs also to be applied. In addition, the use of diagnostic tests has an important value for epidemiological studies and to evaluate the need for intervention measures based on the status of the herds, especially to determine whether there is a situation of enzootic stability or instability. Direct identification of *Babesia* infected-RBC using microscopy has been used traditionally to diagnose bovine babesiosis and is still used today as a practical and inexpensive tool. In the acute period of the disease, the number of parasites in RBC usually increases to a level where they can be readily detected microscopically. This is the case especially for *Babesia* parasites that typically cause high parasitemia, such as *B. bigemina*. Yet, this is not usually the case of acute *B. bovis* infection where infected RBC are hard to find in stained blood films, since they can sequester in large numbers in blood capillaries, in an apparent effort to avoid passing through lymphoid organs, such as the spleen. Additionally, analysis of stained blood films in sub-clinically infected large herds, especially in epidemiological studies, is cumbersome, poorly sensitive, and of limited value. Therefore, more sensitive methods, such as serological assays and molecular tests, should be used. While serological assays are designed to detect immune responses (antibodies) produced by exposed hosts, molecular tests, such as standard PCR, nested-PCR, real-time quantitative PCR, reverse line blot (RLB), and loop-mediated isothermal amplification (LAMP), are aimed at revealing the presence of parasite DNA in the vertebrate host [18,87]. Thus, there is a short window of time at the onset of acute infection (approximately 12–15 days post-infection) when antibodies may not be detected by current serological methods. In turn, this is the stage marked by rapid and unchecked expansion of the parasite in the host, and then, the infection can be promptly detected by molecular methods. However, one possible drawback of PCR methods is the occurrence of false positives due to the persistence of DNA for a short time after effective treatment [101]. Conversely, persistently infected animals may have non-detectable levels of parasite DNA, but detectable levels of circulating anti-*Babesia* antibodies. Therefore, a serological test must be designed based on species-specific antigens that are able to elicit long-term responses and high antibody titers. Yet, serological tests may often have some disadvantages, such as the possible occurrence of poor immune responses in target animals resulting in undetectable levels of antibodies, limited sensitivity of the tests, and cross reactions between parasite species that may turn the tests non-specific. Molecular methods to directly detect the parasite in the field may overcome some of these downsides. RLB has been a preferred method in recent years for the detection of TBD, such as theileriosis, babesiosis, and anaplasmosis, due to its high sensitivity and ability to detect multiple species simultaneously [87]. The use of this technique has allowed the identification of new species and genotypes of *Babesia* parasites worldwide, including Turkey [102,103].

Classic microscopic, serological, and molecular methods have been used for the diagnosis of bovine babesiosis in Turkey [25,26]. Although a set of reliable *Babesia*-diagnostic tests is available nowadays, most of these techniques have disadvantages, such as requiring training and expertise, which makes them difficult to use in field conditions [87]. Alternative rapid and simple point of care strip tests could be developed for this purpose, which requires no previous training for their use, and utilized for the diagnosis of babesiosis, as previously described [104]. Although many studies on strip methods based on immunochromatography or lateral flow test have been done recently [105,106], there is no commercial product available to date worldwide and in Turkey as well, and developing such tests in the future may become important to aid at campaigns designed for the control of bovine babesiosis in this country.

## 7. Current Status of Bovine Babesiosis in Turkey

**Figure 1:**
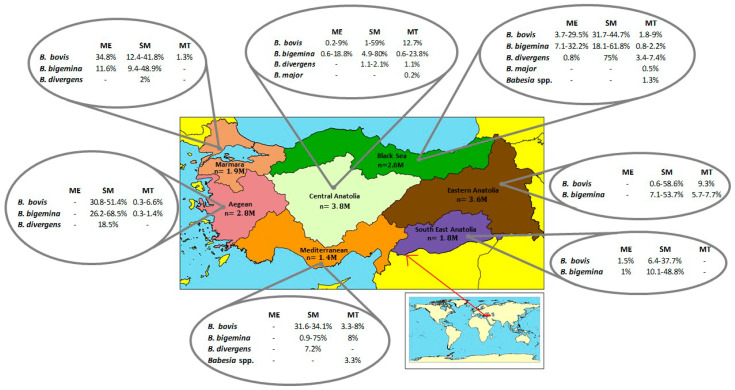
Prevalence rates of bovine *Babesia* species in the seven geographical regions of Turkey according to the microscopic examination (ME), serologic methods (SM), molecular techniques (MT).

Bovine babesiosis has been currently reported in all geographic regions of Turkey. Detection of parasites in the country first began with microscopic description of *B. bigemina* in 1890 [107]. After that, serological studies based on Indirect Fluorescent Antibody Technique (IFAT) revealed exposure to *B. bovis*, *B. bigemina*, and *B. divergens* parasites in different regions of Turkey [108,109,110]. Molecular techniques, such as PCR and RLB, have been recently used to investigate the presence of bovine babesiosis in the country [100,111,112]. Combined serology and molecular results have shown that bovine babesiosis caused by *B. bovis*, *B. bigemina*, *B. divergens*, *B. major*, and *B. occultans* is widespread in several areas of Turkey (Table 2 and Figure 1). Microscopic, serological and molecular methods have shown a wide range prevalence of *Babesia* in cattle in Turkey. The wide range of variation in prevalence described in Table 2 may be due to the use of distinct survey methods with different sensitivities and specificities and/or to real differences among the sets analyzed. These observations underscore the fact that no standardized and systematic survey on bovine babesiosis was so far performed in the country. As a result, the prevalence of bovine babesiosis in Turkey remains unknown. *B. bovis* was first reported by Mimioglu et al. 1969 [113] in cattle in the Black Sea region. Subsequent studies showed that the incidence of *B. bovis* by microscopic analysis ranged between 0.2–9% in the Central Anatolian Region, 3.7–29.5% in the Black Sea Region, 1.5% in the South East Anatolia Region and 34.8% in the Marmara Region. Serological studies focused on *B. bovis* have demonstrated evidence for parasite exposure in herds in all Turkish regions with incidence ranging from 0.6 to 59%. A serological study conducted in 3,773 cattle from all provinces revealed a *B. bovis* prevalence of 34% [110]. In addition, direct assays for *B. bovis* have revealed that the prevalence for *B. bovis* ranged between 0.4–12.7% in Turkey (Table 2 and Figure 1). 

Microscopy analysis revealed that the incidence of *B. bigemina* is 0.6–18.8% in the Central Anatolian Region, 7.1–32.2% in the Black Sea Region, 1% in the South East Anatolia Region, and 11.6% in the Marmara Region. Similar to *B. bovis*, serological assays for *B. bigemina* have shown evidence of parasite exposure (0.9–80%) in all regions in Turkey. A study conducted across the country showed seroprevalence for *B. bigemina* to be 26.3%. The prevalence of *B. bigemina* in molecular studies has been reported at the rate of 0.6–18.2% (Table 2 and Figure 1).

There have been only a few studies performed so far in cattle designed to examine the prevalence of *B. divergens* in Turkey, and the parasite was detected microscopically in only one particular study [100]. Serological studies in cattle reported 75% positivity in the Black Sea region, 7.2% in the Mediterranean region, 18.5% in the Aegean region, and 9.4–48.9% in the Marmara region. *B. divergens* prevalence based on molecular techniques was reported as 3.4–7.4% and 1.1% in the Black Sea and Central Anatolian Regions, respectively (Table 2 and Figure 1).

No positive results have been reported so far regarding *B. major* by microscopy and serological methods in Turkey. In recent years, the molecular diagnostic of *B. major* based on RLB tests has been found a prevalence of 0.2% in the Ankara region [114] and 0.51% in the Eastern Black Sea Region [115] (Table 2 and Figure 1). Similar to *B. major*, only molecular data are available for *B. ocultans* and it was reported a prevalence of 3% for this *Babesia* species using RLB technique in samples from the Black Sea region [100]. In the same study, the authors reported cases of clinical babesisosis caused from *B. bovis*, *B. bigemina*, and *B. divergens* species.

Additional studies have also showed the presence of new uncharacterized *Babesia* genotypes in cattle in Turkey. By using RLB technique, these studies have reported the occurrence of *Babesia* sp. CS58, *Babesia* spp., and *Babesia* sp. *Sivas* in cattle. Blast analysis indicated that their 18S sequences are 99–100% identical to the 18S gene of *B. occultans* [115,116,117] (Table 2). Considering these preliminary findings, further investigation is needed in order to precisely identify these currently uncharacterized *Babesia* organisms in the country and evaluate their implication on the development of bovine babesiosis.

Studies based on molecular methods have reported considerable sequence variations on some *Babesia* genes, such as RAP-1, MSA, and AMA-1. Using a combination of PCR and sequencing techniques, Duzlu et al. (2015) [118] showed that the sequences of the *B. bovis* MSA-2c gene in Turkish isolates are 7.5% different from other isolates in the world. This rate was found to be 0.5% in the *B. bigemina* RAP-1 gene. Another study focused on defining sequence polymorphisms of AMA-1 protein of in vivo and in vitro isolates of “*Babesia bigemina* Kayseri/Turkey” strain. The analysis revealed that nucleotide sequences of Kayseri/Turkey IV1 and Kayseri/Turkey IT2 isolates showed 99.7% identity to each other with a single nucleotide mutation at the position 103. Interestingly, this non-synonymous mutation results in the change of the amino acid at the position 35 (Phe^35^ in Kayseri/Turkey IV1 and Leu^35^ in Kayseri/Turkey IT2) [119]. Implications of this finding need to be investigated in the light of epidemiological and immunopathological view of the disease.

In summary, bovine babesiosis has been reported using different diagnostic methods in all of Turkey’s seven geographical regions (Figure 1). While *B. bovis* and *B. bigemina* are reported in all geographical regions, *B. divergens* has been found more in humid regions located near the seacoast. Despite these many epidemiological investigations, the number of case-based studies is very low. Therefore, prevalence and the actual economic impact of the disease on the livestock industry cannot be accurately assessed, due, in part, to the absence of a national program to systematically record cases of bovine babesiosis in Turkey. Consequently, more well-designed surveys based on highly sensitive assays are needed, especially in areas of the country that so far have received little attention. Although there is no study on annual economic losses associated with bovine babesiosis, the high incidence of the disease in geographical regions that are highly populated by cattle suggests that the disease is already causing serious costs to the livestock industry in Turkey. Clearly, all these findings together strongly suggest the need to organize coordinated efforts aimed at assessing the current status and the development of control measures of bovine babesiosis in Turkey.

## 8. Current Gaps on Bovine Babesiosis Research in Turkey and Suggestions for Intervention

Causative agents of transboundary animal diseases, such as bovine babesiosis, can be easily transported among countries with the increase in intercontinental animal trade [147,148,149]. The number of animals imported to Turkey from various countries has increased in recent years. Animals imported from countries such as Brazil and Australia that are endemic for bovine babesiosis should be tested in order to determine their status before being admitted into Turkey [8,28]. Therefore, there is a need to develop pan-*Babesia* direct and serological assays to be implemented in the country. However, there is a possibility that animals test positive for *Babesia* because they are vaccinated and/or infected with a virulent strain, since vaccination is a common practice in such endemic countries. Regardless of the type of *Babesia* strain infecting these imported animals, they potentially pose an important risk for Turkey’s cattle industry. In these cases, additional practices are needed, such as quarantine to evaluate clinical signs of acute disease and examination for tick infestation, among others. These animal health practices increase the costs for the cattle industry that should be partially or totally covered by government’s funds, or otherwise consumers would have to pay for it. Differentiating infected from vaccinated animals (DIVA) vaccines and diagnostic tests that can also differentiate imported vaccinated from naturally infected animals need to be urgently developed and implemented in the country. Importantly, tick control measurements need also to be performed in such imported animals. Lack of such control in the past resulted in the emergence of *R. microplus*, one of the most devastating vectors of *Babesia* species implicated in bovine babesiosis. Fortunately, *R. microplus* have not been found yet in Turkey, despite the importation of animals from places, such as Brazil and several African countries, where this tick species is endemic [150,151]. *R. microplus* has a major economic impact on milk and beef production in Brazil, and annual losses in this country were estimated at $3.24 billion US dollars [151]. *R. microplus* tends to replace the local boophilic fauna after entering a new region and affects the epidemiology of bovine babesiosis. Rapid spread of *R. microplus* was observed in South Africa, Zambia, and East Africa [5]. Therefore, introduction of *R. microplus* can potentially dramatically change the future status of bovine babesiosis in Turkey. Thus, prevention measures as well as novel research are needed to address gaps in our understanding on the biology and ecology of this tick species in exotic and non-endemic environments. 

Many novel *Babesia* genotypes or species have been recently reported in diverse vertebrates hosts worldwide [6]. Some of these species have also been reported in cattle in Turkey, but no detailed studies on parasite biology and host-parasite-tick interactions have been performed [115,116,117]. While making novel genotype discoveries, the 18S gene region alone has been shown to be insufficient and additional sequencing analysis needs to be performed using other gene regions [5]. The impact, vectors, and pathogenicity of these newly reported genotypes or species of *Babesia* remain largely unknown in Turkey, and these factors need to be further characterized. A recent study in Sri Lanka reported the presence of a novel *Babesia* species, called *Babesia* sp. Mymensingh, which causes clinical babesiosis characterized by fever, hemoglobinuria, anemia, and jaundice in cattle [103,152]. Therefore, studies focused on detailed examination of biology, pathogenicity, host-parasite interactions will fill a large gap that, in turn, can help design strategies to control outbreaks of bovine babesiosis caused by newly, uncharacterized local parasites strains in Turkey.

Use of acaricides is the only method currently used for the prevention of bovine babesiosis in the country. Although there are studies on the efficacy of some acaricides (flumethrin, deltamethrin, cypermethrin) against ticks on cattle in Turkey, investigations on emerging acaricide-resistance in tick populations have not been performed [153,154]. Furthermore, acaricide applications in cattle are usually performed using poorly standardized and unhealthy procedures by breeders in Turkey, and this can potentially generate increased acaricide resistance by ticks due to dose errors as well as environmental and health problems. Livestock producers would benefit greatly from a national training program on the use of acaricides in cattle from the Turkish Ministry of Agriculture. In addition, acaricide applications can also be performed at certain intervals by the local official veterinarians in selected endemic areas. In this way, a more effective tick control program could provide a platform that, in turn, can help decrease the chances for development of acaricide resistance. Economic losses due to TBD can also be reduced by introducing defined policies and requirement for the application of acaricides to livestock, using the frame of existing support programs currently provided by the state, such as the Livestock Support Program.

To date, costs associated with bovine babesiosis in Turkey have not been yet estimated and this analysis is urgently needed. Proper estimation should include cattle mortality, abortions, losses in milk/meat production, loss of draft power, and expenses related to control measures, such as acaricide treatments, and the purchase of therapeutics, among other aspects. In addition, annual losses due to other costs related tick borne diseases (TBD) should also be calculated.

Functional, genetic, and immunological studies on *Babesia* genes and subunit vaccine candidate proteins will be needed in order to develop subunit vaccines, efficient and simpler point of care diagnostic tests, and other possible control methods. Such investigations would be much more relevant to Turkey if local parasites strains are included in comparison with other, more studied strains, such as the virulent T2Bo *B. bovis* strain [155]. Live vaccines are not currently available to prevent bovine babesiosis in the country; therefore, production of live vaccines based on parasites strains that are currently circulating in the country’s provinces should increase control efficacy, prevent outbreaks, and decrease the use of acaricides. Thus, the need for such live vaccines to alleviate the load of bovine babesiosis in Turkey should be addressed and properly assessed.

As a road for future directions on *Babesia* research, well-known and novel, uncharacterized parasite species preserve their mystery like an unconquered land, even after many years of exploration. Numerous characterization studies have been performed up to now and will continue until effective eradication of this parasite is accomplished. Along with genome analysis, important advances have been made in the biology of *Babesia* parasites globally [59,155,156,157]. Afterwards, transfection systems have been developed for gene modification and functional analysis to accelerate vaccine candidate discovery [156]. Together with novel gene editing technologies, these new approaches can potentially accelerate the development of efficient diagnostics and novel vaccine strategies, such as TBV based on *Babesia* sexual-stage antigens. It would be essential to develop a national research program in bovine babesiosis/*Babesia* to translate this new knowledge to the hands of research and field personnel involved in cattle production in Turkey. In vitro tick artificial feeding systems can also be developed and applied to better study tick-*Babesia* interactions [158]. With the development of this system, effectiveness of transstadial and transovarial transmission of many tick-borne pathogens can be determined. Importantly, tick artificial feeding systems can provide a framework for reducing and improving animal use to study ticks and tick-borne pathogens that are important for human and veterinary medicine [158]. Results of such research projects will greatly benefit the development and implementation of control strategies against bovine babesiosis in Turkey.

Subunit vaccines using recombinant antigens produced in *Escherichia coli* expression systems may or may not be efficient in eliciting protective immune responses against *Babesia*. Protection to *Babesia* is believed to be dependent on Th-1 helper cells and early production of IFNγ, which requires adequate antigen presentation by professional antigen-presenting cells via major histocompatibility complex (MHC) class II and effective T cell responses. Perhaps effective subunit vaccines will require the use of mammalian expression, or transfection-based homologous parasite-specific expression systems for antigen production. In addition, as far as vaccine efficacy is concerned, nanoparticles, such as Immune stimulating complexes (ISCOM), are thought to be effective adjuvants for *Babesia* subunit vaccines, which opens new possibilities for the testing of candidate antigens [59].

Novel non-sophisticated point of care diagnostic methods that can be performed by untrained personnel are needed for the rapid “on site” detection of the disease in field conditions and may accelerate the time of response in severe outbreaks of bovine babesiosis. On the other hand, state-of-the-art technologies, such as next-generation and nanopore sequencing approaches, can help our understanding of the composition of local strains, population dynamics of the parasites, and help identify virulence factors that can be used for developing novel vaccines. These techniques combined with gene manipulation techniques already available for the transfection of *Babesia* parasites [156,159] may also lead to better defined and non-tick transmissible live vaccines incorporating DIVA markers to differentiate vaccinated from naturally-infected animals. These DIVA vaccines are much needed in Turkey, especially considering the importation of a large number of live cattle from countries that are endemic for bovine babesiosis. 

Cattle imported into Turkey are currently not subjected to strict sanitary controls, including checking for ticks and TBD, thus imposing an important risk for the development of cattle industries in the country. Such controls need to be incorporated in order to prevent the importation of exotic ticks and *Babesia* strains in the country. As stated before, the introduction of novel tick species that are competent for *Babesia*, such as *R. microplus*, would have devastating consequences, given the ability to these ectoparasites to develop resistance to acaricides, and the lack of alternatives for efficient anti-tick vaccines.

To eliminate the negative effects of acaricides, effective anti-tick vaccine formulations need to be developed based on protective antigens using new generation adjuvants, such as nanoparticles [160]. By combining protective epitopes from different tick proteins into a single chimeric antigen, new vaccines may be likely to target more than one ectoparasite species and host [161]. In addition, microspheres and other sustained-release technologies could also be integrated into acaricides to offer a potential solution for its downside effects. Importantly, new generation sequencing methods can also help identify parasites and other infectious organisms present in distinct tick populations and facilitate our understanding of the interplay among *Babesia* parasites and tick vectors in the ecosystem. Development of new methods for the detection of tick-infested cattle may also help in the control of TBD, including bovine babesiosis.

Collectively, these new technologies and research directions can potentially reveal bottlenecks on the *Babesia* lifecycle and tick ecology that will help implement strategies to control bovine babesiosis to benefit the cattle industry worldwide and particularly in Turkey.

## 9. Concluding Remarks

The world population has been increasing exponentially since the 1950s and nowadays, there is a need to efficiently produce high amounts of animal protein and fiber, and consequently, different human and animal health problems may arise. Protein sources are becoming more valuable every day and an effective and safe method of protection against *Babesia* parasites that affect millions of cattle every year has become more important than ever. Based on the limited data available, it is possible to infer that bovine babesiosis caused by distinct *Babesia* spp. as well as novel and newly identified *Babesia* genotypes/species is highly prevalent throughout Turkey, and safe and effective methods are urgently needed to combat this disease. For this purpose, firstly, multidisciplinary studies need to be performed nationally to address the economic and social impact of bovine babesiosis in Turkey. Secondly, the need for live vaccines should be carefully evaluated, and if needed, vaccines should be produced based on strains of the parasite that are circulating in the country. Use of acaricides, and combinations of these drugs, should be used consciously by technical personnel as part of developed control strategies and practices. In addition, further basic studies on parasite and tick biology need to be performed to develop rationally designed recombinant vaccines. We also should take advantage of newly emerging technologies in order to develop sophisticated tools required to control *Babesia*, a highly co-evolved parasite that is able to evade so efficiently the host immune system and optimize control measures that are currently available. The Turkish cattle industry and, in turn the entire country’s economy, will greatly benefit from such control strategies against the devastating effects of bovine babesiosis. 

## Figures and Tables

**Table 1:** *Babesia* spp. currently identified in cattle with proven vectors and geographical distribution.

Species	Geographical Distribution	Vectors/ITS/TOT	References
*B. bovis* *	tropics, subtropics	*R. annulatus* Ψ/L/+ *R. australis*/L/+ *R. microplus*/L/+	[36,37,38]
*B. bigemina* *	tropics, subtropics	*R. annulatus* Ψ/A/+ *R. australis*/N, A/+ *R. decoloratus*/N, A/+ *R. microplus*/N, A/+ *R. evertsi*/N/− *R. bursa* Ψ/N, A/±	[36,39,40,41]
*B. divergens* *	Europe, North Africa, Russia	*Ixodes ricinus* Ψ/L, N, A/+	[42]
*B. major* *	Europe, North Africa, temperate Asia	*Hae. punctata* Ψ/L, N, A/+	[40]
*B. occultans* *	Africa (Southern Europe, Russia)	*Hy. rufipes* Ψ/A/+	[43]
*B. orientalis*	East Asia	*R. haemaphysaloides*/A/+	[44]
*B. ovata*	East Asia	*Hae. longicornis*/L, N, A/+	[45]

Asterisks (*) and Psi symbols (Ψ) indicate the *Babesia* species and the tick species reported in Turkey respectively. Abbreviations: ITS, Infective Tick Stage; TOT, Transovarial transmission; L, larvae; N, nymph; A, adult.

**Table 2:** Description of *Babesia* spp. present in cattle in Turkey, diagnostic method used in parasite identification, parasite prevalence, and cited references.

Species	Method	Prevelance (%)	Reference
*B. bovis*	ME	0.2–34.8	Göksu 1959 [120]; Göksu 1970 [121]; Cakmak 1987 [108]; Tuzer 1981 [122]; Ozer et al., 1993 [123]; Acıcı 1995 [124]; Inci et al., 2002 [125]; Inci 1992 [126]
	SM	0.6–59	Cakmak 1987 [108]; Sayın et al., 1989 [127]; Dincer et al., 1991; Inci 1992 [126]; Eren 1993 [128]; Sayın et al., 1996 [109]; Ica 2004 [129]; Vatansever et al., 2003 [114]; Aktas et al., 2001 [130]; Inci et al. 2002 [125]; Sayın et al. 1996 [109]; Kalkan et al., 2010 [131]; Oncel et al., 2010 [110]
	MT	0.4–12.7	Tanyüksel et al., 2002 [111]; Bilgin 2007 [132]; Duzlu et al., 2011 [133]; Yavuz et al., 2011 [134]; Aktas and Ozubek 2015 [100]; Duzlu et al., 2015 [118]; Kose et al., 2017 [116]
*B. bigemina*	ME	0.6–32.2	Mimioglu et al., 1955 [135]; Goksu et al., 1959 [120]; Ozcan 1961 [136]; Hoffman et al., 1971 [137]; Tüzer 1981 [122]; Dumanlı and Özer 1987 [138]; Sayin et al., 1989 [127]; Inci 1992 [126]; Ozer et al., 1993 [123]; Acıcı 1995 [124]; Inci et al., 2002 [125]
	SM	0.9–80	Cakmak 1987 [108]; Dincer et al., 1991 [139]; Eren 1993 [128]; Sayın et al., 1996 [109]; Ica 2004 [129]; Vatansever et al., 2003 [114]; Cakmak and Oz 1993 [140]; Vatansever et al., 2001 [141]; Aktas et al., 2001 [130]; Sevinc et al., 2001 [142]; Inci et al., 2002 [125]; Kaya et al., 2006 [143]; Sayın et al., 1996 [109]; Ekici and Sevinc 2009 [33]; Sevgili et al., 2010 [144]; Kalkan et al., 2010 [131]
	MT	0.6–18.2	Tanyuksel et al., 2002 [111]; Ica 2004 [129]; Ica et al., 2007 [145]; Altay et al., 2008 [115]; Duzlu et al., 2011 [133]; Aktas and Ozubek 2015 [100]; Duzlu et al., 2015 [118]; Zhou et al., 2016 [146]
*B. divergens*	ME	0.8	Aktas and Ozubek 2015 [100]
	SM	0.6–75	Sayın et al., 1996 [109]; Aktas et al., 2001 [130]; Inci et al., 2002 [125]
	MT	1.1–7.4	Tanyüksel et al., 2002 [111]; Vatansever et al., 2003 [114]; Aktas and Ozubek 2015 [100]
*B. major*	MT	0.2–0.5	Vatansever et al., 2003 [114]; Altay et al., 2008 [115]
*B. occultans*	MT	3	Aktas et al., 2014 [100]; Aktas and Ozubek 2015 [100]
*Babesia* spp.	MT	1.3	Altay et al., 2008 [115]
*Babesia* spp.	MT	3.3	Kose et al., 2017 [116]
*Babesia* sp. Sivas	MT	1.9	Altay et al., 2020 [117]

Abbreviations: ME, microscopic examination; SM, serologic methods; MT, molecular techniques.
